# Supplementary material for: Preoperative exercise training for adults undergoing elective major vascular surgery: A systematic review
Source: PLoS One. 2022 Jan 26;17(1):e0263090. doi: 10.1371/journal.pone.0263090 (PMC8791536; doi:10.1371/journal.pone.0263090)
Supplement: S1 Table — (DOCX) [file pone.0263090.s001.docx]

**S1 Table. Records that were excluded at the full-text reviewing stage.**

| **Reference** | **Reason for exclusion** |
| --- | --- |
| Janssen TL, Steyerberg EW, van Hoof-de Lepper CC, Seerden TC, de Lange DC, Wijsman JH, Ho GH, Gobardhan PD, van der Laan L. Long-term outcomes of major abdominal surgery and postoperative delirium after multimodal prehabilitation of older patients. Surgery Today. 2020 Nov;50(11):1461-70. | Wrong study design |
| Barberan-Garcia A, Ubré M, Roca J, Lacy AM, Burgos F, Risco R, Momblán D, Balust J, Blanco I, Martínez-Pallí G. Personalised prehabilitation in high-risk patients undergoing elective major abdominal surgery: a randomized blinded controlled trial. Annals of surgery. 2018 Jan 1;267(1):50-6. | Wrong population |
| Partridge JS, Harari D, Martin FC, Peacock JL, Bell R, Mohammed A, Dhesi JK. Randomized clinical trial of comprehensive geriatric assessment and optimization in vascular surgery. Journal of British Surgery. 2017 May;104(6):679-87. | Wrong intervention |
| Mifsud M, Cassar K. The use of transcutaneous electrical stimulation of the calf in patients undergoing infrainguinal bypass surgery. Annals of vascular surgery. 2015 Nov 1;29(8):1524-32. | Wrong intervention |
| Mohamady HM, Waked IS, Attalla AF. Preoperative respiratory physical therapy program as a prehabilitation to improve inspiratory muscle function and quality of life in patients undergoing upper abdominal surgeries: a prospective randomized controlled trial. Bulletin of Faculty of Physical Therapy. 2016 Jun;21(1):17-22. | Wrong population |
| Boden IJ. Preoperative physiotherapy to prevent postoperative pulmonary complications after major abdominal surgery (Doctoral dissertation). | Wrong population |
| Haque AA. Exercise and fitness technologies to improve outcomes for vascular patients (Doctoral dissertation). | Wrong population |
| Roukema JA, Carol EJ, Prins JG. The prevention of pulmonary complications after upper abdominal surgery in patients with noncompromised pulmonary status. Archives of Surgery. 1988 Jan 1;123(1):30-4. | Wrong population |
| Chumillas S, Ponce J, Delgado F, Viciano V, Mateu M. Prevention of postoperative pulmonary complications through respiratory rehabilitation: a controlled clinical study. Archives of physical medicine and rehabilitation. 1998 Jan 1;79(1):5-9. | Wrong population |
| Celli BR, Rodriguez KS, Snider GL. A controlled trial of intermittent positive pressure breathing, incentive spirometry, and deep breathing exercises in preventing pulmonary complications after abdominal surgery. American Review of Respiratory Disease. 1984 Jul;130(1):12-5. | Wrong population |
| Soares SM, Nucci LB, da Silva MM, Campacci TC. Pulmonary function and physical performance outcomes with preoperative physical therapy in upper abdominal surgery: a randomized controlled trial. Clinical rehabilitation. 2013 Jul;27(7):616-27. | Wrong population |
