# Supplementary material for: Preoperative exercise training for adults undergoing elective major vascular surgery: A systematic review
Source: PLoS One. 2022 Jan 26;17(1):e0263090. doi: 10.1371/journal.pone.0263090 (PMC8791536; doi:10.1371/journal.pone.0263090)
Supplement: S2 Table — (DOCX) [file pone.0263090.s002.docx]

**S2 Table. Descriptions of the exercise components used in prehabilitation interventions according to Consensus Exercise Reporting Template (CERT).**

| **Section/Topic** | **Item #** | **Abbreviated Item Description** | **Dronkers 2008** | **Barakat 2016** | **Tew 2017** |
| --- | --- | --- | --- | --- | --- |
| WHAT: materials | 1 | Type of exercise equipment | An inspiratory muscle training device (Threshold Inspiratory Muscle Training; Respironics, Pittsburgh, PA, USA). | Cycle ergometer, treadmill, dumbbells | A cycle ergometer (Optibike Med; Ergoline, Bitz, Germany). |
| WHO: provider | 2 | Qualifications, teaching/supervising expertise and/or training of the exercise instructor | One session per week was supervised by the same physical therapist. The therapist was described as “experienced”, but their specific qualifications and expertise was not stated. | Not reported | “Each session was supervised directly by a research nurse and a physiotherapist who were trained in immediate life support… Sessions were also attended intermittently by one of two experienced exercise scientists…” |
| HOW: delivery | 3 | Whether exercises were performed individually or in a group | Not reported | Group-based; group size not reported | Not reported |
|  | 4 | Whether exercises were supervised or unsupervised and how they were delivered | One session per week was directly supervised and five sessions per week were unsupervised. | All sessions were directly supervised | All sessions were directly supervised |
|  | 5 | How adherence to exercise was measured and reported | Self-reported in a diary | Class attendance was documented | Each session was documented by the physiotherapist. They also recorded the following for each exercise interval: heart rate, perceived exertion, blood pressure, and power output. |
|  | 6 | Details of motivation strategies used | None reported | “… maintaining continuous clinical supervision throughout the study to provide appropriate training, advice, and encouragement while ensuring participants’ well being.” | None reported |
|  | 7 | How and when the exercise programme is progressed | The resistance was increased incrementally, based on the rate of perceived exertion (RPE) scored by the patient on the Borg Scale. If the RPE was <5, the resistance of the training device was increased incrementally by 2 cmH_2_O. | Not reported | From the second session onwards, the cycling power output was guided by the participant’s RPE responses, which were assessed separately for legs (RPE-L) and breathlessness/ chest (RPE-C) at the end of each interval using Borg's CR-10 scale. The aim was for all work intervals to be undertaken at a hard to very hard level of exertion (RPE-L or RPE-C of 5 and 7, respectively). |
|  | 8 | Detailed description of each exercise to enable replication? | The inspiratory muscle training protocol is described in sufficient detail to allow replication. | The description of the circuit-based exercise classes lacked information on intensity and progression. | The cycling protocol is described in sufficient detail to allow replication. |
|  | 9 | Content of any home programme component | Participants performed five sessions per week of unsupervised inspiratory muscle training. These unsupervised sessions had the same content as the weekly supervised session. | Not applicable | Not applicable |
|  | 10 | Non-exercise components | None reported | None reported | None reported |
|  | 11 | How exercise-related adverse events were documented and managed | Adverse events were self-reported in a diary. No procedures for managing adverse events were reported. | Not reported | Adverse events were recorded in a local adverse event log. Events were classified according to severity and whether they were related to the study intervention. The following control measures were implemented: thorough preparticipation screening; exclusion of high-risk patients; hospital-based exercise testing and training; exercise sessions supervised by experienced cardiac physiotherapists trained in Intermediate Life Support; exercise sessions performed >3 h after waking given the higher frequency of cardiovascular events during the morning hours; prompt evaluation of prodromal symptoms; resuscitation equipment and oxygen immediately available; exercise termination if a patient has signs/symptom suggestive of distress, cardiac or aneurysm-related adverse event; reduction in cycling power output if a participant’s systolic blood pressure was >180 mmHg, or heart rate was >95% of the peak heart rate recorded on the baseline exercise test. |
| WHERE: location | 12 | Setting in which the exercises are performed | Gelderse Vallei Hospital Ede in the Netherlands was specified as the study setting; however, it was not explicitly stated that this was where the supervised sessions were performed. | Exercise classes were delivered in a hospital-based physiotherapy department. Participants joined an established local exercise programme that was originally designed for people with intermittent claudication. | Exercise sessions were conducted in three teaching hospitals in England: James Cook University Hospital, Middlesbrough; Northern General Hospital, Sheffield; and York Hospital, York. |
| WHEN, HOW MUCH: dosage | 13 | Detailed description of the exercise intervention (e.g., reps, sets, intensity, session frequency and duration, programme duration) | “The intervention group took part in a training programme (six sessions, six days a week for at least two weeks before surgery) designed to increase the strength and endurance of the inspiratory muscles. Each session consisted of 15 minutes of inspiratory muscle training… The subjects started breathing at a resistance equal to 20% of their maximal inspiratory pressure, measured at baseline, for 15 minutes a day. The resistance was increased incrementally, based on the rate of perceived exertion (RPE) scored by the patient on the Borg Scale. If the RPE was <5, the resistance of the inspiratory threshold trainer was increased incrementally by 2 cmH_2_O.” | “Patients allocated to the intervention arm, that is, exercise, were provided with instructions and a time table to join hospital based exercise classes, carried out 3 times a week, for 1-hour duration, in the physiotherapy gym. The scheduled exercise program was for a total of 6 consecutive weeks immediately preceding the intended operation date. Each exercise class consisted of the following: 5-minute warm up and stretching, cycle ergometer against moderate resistance for 2 minutes, heel-raise repetitions for 2 minutes, knee extensions against resistance repetitions for 2 minutes, dumbbells’ biceps/arm curls repetitions for 2 minutes, step-up lunges repetitions for 2 minutes, knee bends (bodyweight) repetitions for 2 minutes, and 5 minutes for cool down and stretching. Between each of the exercise stations, patients either walked around the gym or on a treadmill, or rested for 2 minutes before moving on to the next exercise.” | “Participants allocated to the exercise group were also invited to complete three hospital-based exercise sessions per week, for the 4 consecutive weeks (weeks 1–4; main phase) immediately preceding their intended operation date (in week 5). Participants whose operation was delayed beyond week 5 (for example owing to lack of availability of a hospital bed) also received a maintenance phase of training (1 exercise session per week). All exercise was undertaken on a cycle ergometer (Optibike Med; Ergoline, Bitz, Germany). Each of the first three sessions comprised a 10-min warm-up of unloaded cycling, eight 2-min intervals of high-intensity cycling interspersed with 2-min rest periods of unloaded cycling, and then a 5-min cool-down of unloaded cycling. In all subsequent sessions, participants had the choice of performing eight 2-min or four 4-min ‘work’ intervals for the main body of the workout. In the first exercise session, the 2-min work intervals were performed at the power output corresponding to anaerobic threshold on a baseline cardiopulmonary exercise test (CPET). The power output in all subsequent sessions was guided by participants' ratings of perceived exertion (RPE), which were assessed separately for legs (RPE-L) and breathlessness/chest (RPE-C) at the end of each interval using Borg's CR-10 scale. The aim was for all work intervals to be undertaken at a hard to very hard level of exertion (RPE-L or RPE-C of 5 and 7 respectively).” |
| TAILORING: what, how | 14a | Whether exercises were generic (“one size fits all”) or tailored to the individual | Tailored to the individual | Not reported | Tailored to the individual |
|  | 14b | How exercises were tailored to the individual | The starting resistance was based on the individual’s maximal inspiratory pressure, and progression was based on the individual’s RPE responses. | Not reported | The starting power output was based on the individual’s anaerobic threshold, and progression was based on the individual’s RPE, blood pressure, and heart rate responses. |
|  | 15 | Decision rules for determining the starting level of exercise | Participants started breathing at a resistance equal to 20% of their maximal inspiratory pressure, measured at baseline. | Not reported | “In the first exercise session, the 2-min work intervals were performed at the power output corresponding to anaerobic threshold on a baseline cardiopulmonary exercise test (CPET).” |
| HOW WELL: planned, actual | 16a | How intervention fidelity was assessed | Participants completed a training diary. There was no detail on what exactly they were asked to record. | Class attendance was documented | Each session was documented by the physiotherapist. They also recorded the following for each exercise interval: heart rate, perceived exertion, blood pressure, and power output. “Sessions were also attended intermittently by one of two experienced exercise scientists, who had overall responsibility for ensuring treatment fidelity of the exercise programme.” |
|  | 16b | The extent to which the intervention was delivered as planned | “All participants reported their daily inspiratory muscle training workout in their diaries.” | 11 (17.7%) participants did not attend any classes, 19 (30.6%) attended 6-12 classes, and 32 (51.6%) attended 13-18 classes. | 240/324 (74.1%) main-phase sessions and 36/40 (90%) were completed, giving an overall attendance rate of 75·8%. 17/27 (63%) participants achieved the prespecified adherence criterion of completing at least 9 out of 12 main-phase sessions and all maintenance sessions. “The intensity of all work intervals completed by the 17 adherent participants is summarized as follows: mean(s.d.) RPE-L 4·1(2·0), RPE-C 3·5(1·9) and heart rate 81·7(8·5) per cent maximum. Some 30 per cent of work intervals were reported in the hard to very hard range (RPE-L 5–7).” |
