# Supplementary material for: Preoperative exercise training for adults undergoing elective major vascular surgery: A systematic review
Source: PLoS One. 2022 Jan 26;17(1):e0263090. doi: 10.1371/journal.pone.0263090 (PMC8791536; doi:10.1371/journal.pone.0263090)
Supplement: S1 File — (DOCX) [file pone.0263090.s003.docx]

**S1 Text. Database search strategies**

**MEDLINE (Ovid)**

1 Prehab*.mp.

2 Pre-hab*.mp.

3 Preop*.mp.

4 Pre-op*.mp.

5 Presurg*.mp.

6 Pre-surg*.mp.

7 1 or 2 or 3 or 4 or 5 or 6

8 Randomized controlled trial.m_titl.

9 Controlled clinical trial.m_titl.

10 randomized.ab.

11 placebo.ab.

12 drug therapy.fs.

13 randomly.ab.

14 trial.ab.

15 groups.ab.

16 8 or 9 or 10 or 11 or 12 or 13 or 14 or 15

17 exp animals/ not humans.sh.

18 16 not 17

19 exp Vascular Diseases/

20 exp Vascular Surgical Procedures/

21 19 or 20

22 7 and 18 and 21

23 limit 22 to yr="2008 -Current"

**Embase (Ovid)**

1 Prehab*.mp.

2 Pre-hab*.mp.

3 Preop*.mp.

4 Pre-op*.mp.

5 Presurg*.mp.

6 Pre-surg*.mp.

7 1 or 2 or 3 or 4 or 5 or 6

8 Randomized controlled trial/

9 Controlled clinical trial/

10 random$.ti,ab.

11 randomization/

12 intermethod comparison/

13 placebo.ti,ab.

14 (compare or compared or comparison).ti.

15 ((evaluated or evaluate or evaluating or assessed or assess) and (compare or compared or comparing or comparison)).ab.

16 (open adj label).ti,ab.

17 ((double or single or doubly or singly) adj (blind or blinded or blindly)).ti,ab.

18 double blind procedure/

19 parallel group$1.ti,ab.

20 (crossover or cross over).ti,ab.

21 ((assign$ or match or matched or allocation) adj5 (alternate or group$1 or intervention$1 or patient$1 or subject$1 or participant$1)).ti,ab.

22 (assigned or allocated).ti,ab.

23 (controlled adj7 (study or design or trial)).ti,ab.

24 (volunteer or volunteers).ti,ab.

25 human experiment/

26 trial.ti.

27 8 or 9 or 10 or 11 or 12 or 13 or 14 or 15 or 16 or 17 or 18 or 19 or 20 or 21 or 22 or 23 or 24 or 25 or 26

28 (random$ adj sampl$ adj7 (cross section$ or questionnaire$1 or survey$ or database$1)).ti,ab. not (comparative study/ or controlled study/ or randomi?ed controlled.ti,ab. or randomly assigned.ti,ab.)

29 Cross-sectional study/ not (randomized controlled trial/ or controlled clinical study/ or controlled study/ or randomi?ed controlled.ti,ab. or control group$1.ti,ab.)

30 (((case adj control$) and random$) not randomi?ed controlled).ti,ab.

31 (Systematic review not (trial or study)).ti.

32 (nonrandom$ not random$).ti,ab.

33 Random field$.ti,ab.

34 (random cluster adj3 sampl$).ti,ab.

35 (review.ab. and review.pt.) not trial.ti.

36 we searched.ab. and (review.ti. or review.pt.)

37 update review.ab.

38 (databases adj4 searched).ab.

39 (rat or rats or mouse or mice or swine or porcine or murine or sheep or lambs or pigs or piglets or rabbit or rabbits or cat or cats or dog or dogs or cattle or bovine or monkey or monkeys or trout or marmoset$1).ti. and animal experiment/

40 Animal experiment/ not (human experiment/ or human/)

41 28 or 29 or 30 or 31 or 32 or 33 or 34 or 35 or 36 or 37 or 38 or 39 or 40

42 27 not 41

43 exp vascular disease/

44 exp vascular surgery/

45 43 or 44

46 7 and 42 and 45

47 limit 46 to yr="2008 -Current"

**CINAHL Complete (EBSCO)**

S1 TX Prehab*

S2 TX Pre-hab*

S3 TX Preop*

S4 TX Pre-op*

S5 TX Presurg*

S6 TX Pre-surg*

S7 (S1 OR S2 OR S3 OR S4 OR S5 OR S6)

S8 MH randomized controlled trials

S9 MH double-blind studies

S10 MH single-blind studies

S11 MH random assignment

S12 MH pretest-posttest design

S13 MH cluster sample

S14 TI (randomised OR randomized)

S15 AB (random*)

S16 TI (trial)

S17 MH (sample size)

S18 AB (assigned OR allocated OR control)

S19 S17 AND S18

S20 MH (placebos)

S21 PT (randomized controlled trial)

S22 AB (control W5 group)

S23 MH (crossover design)

S24 MH (comparative studies)

S25 S23 OR S24

S26 AB (cluster W3 RCT)

S27 MH animals+

S28 MH (animal studies)

S29 TI (animal model*)

S30 S27 OR S28 OR S29

S31 MH (human)

S32 S30 NOT S31

S33 S8 OR S9 OR S10 OR S11 OR S12 OR S13 OR S14 OR S15 OR S16 OR S19 OR S20 OR S21 OR S22 OR S25 OR S26

S34 S33 NOT S32

S35 (MH "Vascular Diseases+")

S36 (MH "Vascular Surgery+")

S37 S35 OR S36

S38 S7 AND S34 AND S37

**CENTRAL**

#1 (prehab*):ti,ab,kw

#2 (pre-hab*):ti,ab,kw

#3 (preop*):ti,ab,kw

#4 (pre-op*):ti,ab,kw

#5 (presurg*):ti,ab,kw

#6 (pre-surg*):ti,ab,kw

#7 #1 OR #2 OR #3 OR #4 OR #5 OR #6

#8 MeSH descriptor: [Vascular Diseases] explode all trees

#9 MeSH descriptor: [Vascular Surgical Procedures] explode all trees

#10 #8 OR #9

#11 #7 AND #10 with Publication Year from 2008 to present, in Trials
