# Supplementary material for: Preoperative exercise training for adults undergoing elective major vascular surgery: A systematic review
Source: PLoS One. 2022 Jan 26;17(1):e0263090. doi: 10.1371/journal.pone.0263090 (PMC8791536; doi:10.1371/journal.pone.0263090)
Supplement: Attachment — Submitted filename: Response to Reviewers.docx [file pone.0263090.s004.docx]

**Response to Reviewers**

**Preoperative exercise training for adults undergoing elective major vascular surgery: A systematic review**

**Journal Requirements:**

**1. Please ensure that your manuscript meets PLOS ONE's style requirements, including those for file naming. The PLOS ONE style templates can be found at**

[**https://journals.plos.org/plosone/s/file?id=wjVg/PLOSOne_formatting_sample_main_body.pdf**](https://journals.plos.org/plosone/s/file?id=wjVg/PLOSOne_formatting_sample_main_body.pdf) **and**

[**https://journals.plos.org/plosone/s/file?id=ba62/PLOSOne_formatting_sample_title_authors_affiliations.pdf**](https://journals.plos.org/plosone/s/file?id=ba62/PLOSOne_formatting_sample_title_authors_affiliations.pdf)**.**

We have reformatted the headings and updated the figure citations and file names for figures. Supporting information has been uploaded separately as individual files. Postal codes have been removed from affiliations.

**2. Thank you for stating the following in the Competing Interests section:**

**[I have read the journal's policy and the authors of this manuscript have the following competing interests: GT and GD were investigators on one of the included trials (Tew 2017).]**

We have re-named this section as “Competing interests” and inserted the following variant of what has been suggested: “The authors of this manuscript have the following competing interests: GT and GD were investigators on one of the included trials (Tew 2017).”

**Please confirm that this does not alter your adherence to all PLOS ONE policies on sharing data and materials, by including the following statement: "This does not alter our adherence to PLOS ONE policies on sharing data and materials.” (as detailed online in our guide for authors http://journals.plos.org/plosone/s/competing-interests). If there are restrictions on sharing of data and/or materials, please state these. Please note that we cannot proceed with consideration of your article until this information has been declared.**

We have included that statement in the Competing Interests section.

**Please include your updated Competing Interests statement in your cover letter; we will change the online submission form on your behalf.**

The updated statement has been included in our cover letter.

**3. Please review your reference list to ensure that it is complete and correct. If you have cited papers that have been retracted, please include the rationale for doing so in the manuscript text, or remove these references and replace them with relevant current references. Any changes to the reference list should be mentioned in the rebuttal letter that accompanies your revised manuscript. If you need to cite a retracted article, indicate the article’s retracted status in the References list and also include a citation and full reference for the retraction notice.**

We have reviewed the reference list and believe it to be complete and correct.

**Additional Editor Comments:**

**Thank you for submitting this interesting article. Some comments have been made by the expert Reviewers. Please address the comments raised if you are considering a re-submission.**

We are glad that you think our article is interesting. We have addressed all review comments.

**Reviewers' comments:**

**Reviewer's Responses to Questions**

**Comments to the Author**

**1. Is the manuscript technically sound, and do the data support the conclusions?**

**The manuscript must describe a technically sound piece of scientific research with data that supports the conclusions. Experiments must have been conducted rigorously, with appropriate controls, replication, and sample sizes. The conclusions must be drawn appropriately based on the data presented.**

**Reviewer #1: Yes**

**Reviewer #2: Yes**

**2. Has the statistical analysis been performed appropriately and rigorously?**

**Reviewer #1: Yes**

**Reviewer #2: Yes**

**3. Have the authors made all data underlying the findings in their manuscript fully available?**

**The PLOS Data policy requires authors to make all data underlying the findings described in their manuscript fully available without restriction, with rare exception (please refer to the Data Availability Statement in the manuscript PDF file). The data should be provided as part of the manuscript or its supporting information, or deposited to a public repository. For example, in addition to summary statistics, the data points behind means, medians and variance measures should be available. If there are restrictions on publicly sharing data—e.g. participant privacy or use of data from a third party—those must be specified.**

**Reviewer #1: Yes**

**Reviewer #2: Yes**

**4. Is the manuscript presented in an intelligible fashion and written in standard English?**

**PLOS ONE does not copyedit accepted manuscripts, so the language in submitted articles must be clear, correct, and unambiguous. Any typographical or grammatical errors should be corrected at revision, so please note any specific errors here.**

**Reviewer #1: Yes**

**Reviewer #2: Yes**

**5. Review Comments to the Author**

**Please use the space provided to explain your answers to the questions above. You may also include additional comments for the author, including concerns about dual publication, research ethics, or publication ethics. (Please upload your review as an attachment if it exceeds 20,000 characters)**

**Reviewer #1: For transparency to the authors i have already reviewed this document and provided feedback which i believe has mainly been addressed. However i did not see the previous response to reviewer document. Some further considerations for review:**

We appreciate the time you have taken to review our manuscript and thank you for the thoughtful and helpful comments.

**1. With regard to the RoB tool, i would consider an external reviewer as some of the papers are the authors own.**

The risk of bias assessments were conducted independently by two reviewers. One of the reviewers (KC) was not involved in the any of the included studies. There were no discrepancies in two reviewers’ judgements, so we are confident that our assessments are appropriate and unbiased.

**2. Consider a secondary search as the current one is nearing 8 months post completion.**

We performed a rerun of the database searches on December 7, 2021. No further relevant RCTs were identified.

**3. This review only included 3 studies but a recently published Cochrane - identifies 4 (https://pubmed.ncbi.nlm.nih.gov/34236703/) please consider whether the missing study should be included in this review?**

We think that the reviewer is referring to the following pilot study of Richardson and colleagues: <https://clinicaltrials.gov/ct2/show/NCT02845167>. This study was excluded during our screening process because the intervention involved exercise sessions on three consecutive days. For inclusion in our review, the training duration needed to be at least one week.

**4. Regarding the complications in the Baraket paper these were found to be surgery specific OPEN vs EVAR, it would be good to demonstrate this in the results section.**

We have added the procedure-specific complication rates to Table 3, as well as the following sentence: “An exploratory (i.e., underpowered) sub-group analysis indicated that the effect was procedure-specific, with a greater risk reduction being observed in patients undergoing open surgical repair rather than endovascular repair.”

**5. Page 15 lines 222-223 & 227-228 are repeats.**

These sentences are worded similarly, but both are relevant and worthy of inclusion as they relate to different outcomes. The first sentence refers to how we handled the mortality data. The second sentence refers to how we handled the complications data.

**6. Please discuss the discrepancy between the RoB reported here as low but reported as very high in the Cochrane review.**

The Cochrane review of Fenton et al. (2021) used version 1 of the Cochrane RoB tool, whereas we used version 2, so the RoB results are not directly comparable. We can however comment on some apparent discrepancies. First, we did not consider there to be high risk of bias due to selective reporting because data were presented for the outcome measures that we were interested in. Second, Fenton et al. gave the study of Tew et al. a rating of ‘high risk’ for incomplete outcome data, stating that attrition was >20% and ITT analysis wasn’t used. Both these statements are incorrect. Third, in ‘other potential sources of bias’, Fenton et al. gave a high risk of bias rating to the study of Tew et al. for it being underpowered. This goes against Cochrane guidance that criteria related to precision should not be assessed within this domain (<https://handbook-5-1.cochrane.org/chapter_8/8_15_2_assessing_risk_of_bias_from_other_sources.htm>). Finally, we would like to note that that RoB results in the NICE evidence review (reference #17) are in keeping with our results.

**7. Due to the lack of studies found in PAD and carotid disease, this review does have an overlap with the published Cochrane review. Is there consideration to expand your review to non-RCTs to include more data, although acknowledging that this is not as "high level evidence".**

Quasi-RCTs were eligible for inclusion, however no such studies were found. We decided not to include uncontrolled studies because they provide little insight into the quantitative effect of prehabilitation on post-operative outcomes. Limiting the inclusion of studies to RCTs also reflects the approach used in evidence syntheses to inform clinical guidelines (i.e., to focus on “high-level evidence).

**Reviewer #2: This is well-conducted systematic review of the published evidence regarding pre-habilitation exercise programmes prior to major vascular surgery (AAA, Lower limb bypasses and Carotid endarterectomy). Unfortunately there were only 3 RCTs which fulfilled the inclusion criteria, however this reflects the current lack of evidence in this area. The manuscript is well-written, however could benefit from shortening of the discussion section, where there seems to be a repetition of some of the findings in the results section.**

Thank you for reviewing our manuscript. We have carefully reviewed the discussion section and would prefer not to shorten it. This is because we think that all the content is valuable and most of it is new (i.e., not repeated). For example, the discussion around implications for policy, practice and research and the strengths and limitations, which makes up most of the text, is all unique to this section. Also, at 1,828 words, the size of this section is in keeping with the rest of the manuscript.
